# Does intracranial pressure management hurt more than it helps in traumatic brain injury?

**DOI:** 10.1136/tsaco-2017-000142

**Published:** 2018-01-12

**Authors:** Charles A Adams, Deborah M Stein, Jonathan J Morrison, Thomas M Scalea

**Affiliations:** 1 Division of Trauma and Critical Care, Rhode Island Hospital, Brown University, Providence, Rhode Island, USA; 2 R Adams Cowley Shock Trauma Center, University of Maryland, Baltimore, Maryland, USA

**Keywords:** tbi, icp, traumatic brain injury, intracranial pressure

## Abstract

Traumatic brain injury (TBI) is the leading cause of death after traumatic injury. Raised intracranial pressure (ICP) is particularly associated with poor TBI outcomes, prompting clinicians to monitor this parameter, using it to guide therapies aimed at reducing pressures. Despite this approach being recommended by several bodies such as the Brain Trauma Foundation and the American College of Surgeons, the evidence demonstrating that ICP-guided therapy improves outcome is limited. The topic was debated at the 36th Annual Point/Counterpoint Acute Care Surgery Conference and the following article summarizes the discussants points of view along with a summary of the evidence.

**Level of evidence:**

Level III.

## Introduction

Traumatic brain injury (TBI) is the leading cause of death from trauma, affecting 10 million people annually worldwide.^1^ Survival is often complicated by functional limitation, incurring significant personal, familial and societal cost. In the USA, TBI accounts for 50 000 deaths a year, with 235 000 attributed hospital admissions.^2^

The foundation which has guided the management principles for TBI was established over two centuries ago by the Scottish physicians Alexander Munro and George Kellie.^3^ After refinement, their doctrine is based on the premise that the cranial vault is a fixed volume, containing brain parenchyma (80%), cerebrospinal fluid (10%) and blood (10%). Should the primary injury result in a focal (eg, hematoma) or diffuse (eg, parenchymal swelling) space occupying lesion (SOL), intracranial pressure (ICP) will increase, potentially compromising one or more of these components.

The mechanisms and consequences of raised ICP are multifactorial and complex, but simplistically, the brain can reduce cerebrospinal fluid volume to compensate for a modest SOL, helping to maintain a ‘normal’ ICP. However, once that compensatory threshold has been exceeded, the brain will decompensate with compression of brain parenchyma and reduced cerebral perfusion pressure (CPP). This spiral results in secondary brain injury and if unabated, can be fatal.

Numerous retrospective clinical studies have associated raised ICP with poor outcome, and there has been a general assumption that treating ICP will, therefore, improve outcome. As a consequence, the monitoring of ICP to direct optimum treatment is conceptually attractive; however, the evidence is subject to debate. This article is a summary of the debate on this subject held during the 36th Annual Point/Counterpoint Acute Care Surgery Conference.

## **ICP management does not hurt more than it helps** (abridged summary)

**Dr Deborah M Stein, MD, Professor of Surgery, R Adams Cowley Shock Trauma Center**

Modern management of TBI is targeted towards the prevention and mitigation of secondary brain insults such as the prevention of systemic hypotension and hypoxia. ICP monitoring has become customary in the modern ICU, but is largely based on retrospective data.

The Brain Trauma Foundation’s (BTF) most recent guidelines were published in 2016 and recommend ICP monitoring to ‘…reduce in-hospital and 2 week post-injury mortality…’. What remains to be analyzed is exactly what patient population needs to be monitored, for how long and in what form.

ICP monitoring is the most rapid and consistent methods for identifying cerebral edema. It can also give advanced warning of SOL expansion, venous outflow compromise, loss of autoregulation, among other important neurological events. Although other methods do exist, such as serial CT scans and pauses in sedation to allow for neurological assessment, these methods are associated with real risks such as radiation exposure and altered physiology which can adversely affect functional outcome.

The only randomized, controlled trial (RCT) to address the issue of whether ICP monitoring changes outcome failed to demonstrate a difference between groups. There are several important methodological issues which affect the interpretation and generalizability of this trial (which will be discussed in detail later); however, and importantly, there was no specific harm associated with ICP monitoring.

## **ICP management does hurt more than it helps** (abridged summary)

**Dr Charles A Adams, MD, Associate Professor of Surgery, Rhode Island Hospital**

Despite ICP measurement being presented as a central tenant of multiple evidence-based TBI guidelines, the evidence largely consists of retrospective studies and a single RCT performed in a settling not generalizable to the USA. Furthermore, although the Munro-Kellie doctrine appears mechanistically straightforward, the interplay between injury and intervention is far more complex.

When considering harm, it is important to note that ICP monitoring per se does not lead to adverse events, but rather it is the care that is driven from such monitoring that is the consequence. These management efforts are motivated by a desire to maintain CPP, which can result in the use of aggressive interventions such as mannitol or hypertonic saline. Some interventions are known to be harmful to certain groups, such as the elderly, which have limited cardiovascular and/or renal reserve. Decompressive craniectomy is another example of an aggressive intervention, performed in response to raised ICP, which can lead to the improved survival of patients with little to no chance of a functional life.

In considering this issue further, practice patterns often provide clues as to practicing clinicians’ views on the value of an intervention. Despite clear guidelines on the use of ICP monitoring, less than half of patients with an indication for ICP monitoring, actually receive one. This may reflect the intuition of the neurosurgeons that ICP monitors add little to the care of TBI.

The use of ICP monitoring has a poor evidence base, and it is clear that it can drive some interventions that are associated with harmful morbidity. This issue requires more thorough evaluation before it should be considered a standard of care.

## Evidence summary

The first report of ICP monitoring was described by Lundberg and colleagues in 1964, where they implanted a ventricular cannula in 30 patients with TBI.^4^ In addition to a description of the observed physiology, they hypothesized that ICP monitoring may form a rational basis for treatment, rather than current metrics, which were largely based on clinical observation. This article has set the stage for the next 40 years of ICP-directed TBI management.

During the following two decades, several teams have demonstrated the utility of ICP in the prognostication of TBI. Miller *et al* from Virginia used ICP monitoring in 160 patients with severe TBI and identified that a threshold of 20 mm Hg was associated with a particularly poor outcome.^5^ Interestingly, in half of the patients with an ICP >20 mm Hg, this proved refractory to interventions considered state-of-the-art at the time (ventilation and steroids). This same group were also able to stratify outcome by ICP, with a mortality from a normal ICP, raised and refractory reported as 18%, 26% and 92%.^6^ This group also associated these findings with Glasgow Coma Scale and ocular response.

In parallel with these findings, the question soon arose as to whether ICP monitoring could be used as a therapeutic target to improve TBI outcomes.^7^ One of the first uses of ICP monitoring was in conjunction with CT scanning in the role of intervention in occult SOL.^8^ The group in Glasgow was able to demonstrate that in the absence of a clear indication for operative intervention, ICP monitoring could be used to monitor patients, successfully identifying the minority that eventually required craniotomy.

However, ICP monitoring has largely used to direct active intervention such as hyperosmolar therapy, decompressive craniectomy, sedation and ventilator strategies. One of the largest studies comparing ICP-monitored patients to those without has come from the ACS Trauma Quality Improvement Program (TQIP) which analyzed 10 628 patients with severe TBI.^9^ Although ICP monitoring was only used in 17.6% of patients, it was associated with a substantial reduction in mortality: OR (95% CI) of 0.44 (0.31 to 0.63). This difference was examined in greater detail, assessing the case mix and institutional volume, but after modeling, the benefit persisted.

Evidence, such as the TQIP study, is regularly reviewed and synthesized into guidelines by organizations such as the BTF, which recommends the use of ICP monitoring in severe TBI and nationally is considered a standard of care. However, it is acknowledged that the evidence base is largely level 3, based on retrospective observational data, which carries intrinsic limitation. Curiously, the most recent recommendations have removed some of the explicit direction, which may represent acknowledgement that the issue is open to interpretation.

Despite these guidelines, compliance is variable, which does appear to affect outcome. A multicenter retrospective study of 11 US level1 trauma centers demonstrated that 76% of institutions followed ICP-directed therapy guidelines.^10^ When the authors went on to control for additional variables, it was found that overall compliance with evidence-based TBI management (endotracheal intubation, resuscitation, etc, in additional to ICP monitoring) was associated with superior outcomes (OR 0.88; 95% CI 0.81 to 0.96, P<0.005).

These findings were further reinforced by a subsequently propensity-score matched study from the USA which compared the outcomes of ICP-monitored patients to those without.^11^ First, they demonstrated that only 46% of eligible patients actually received an ICP monitor, which has led some to suggest this is reflective of neurosurgical pessimism towards ICP monitoring. However, this is speculative and it is conceivable they were not used due to reservations regarding poor prognosis, as the unadjusted mortality was higher in the unmonitored group. The more significant finding was that the use of ICP monitoring in matched TBI cohorts demonstrated an 8.3% reduction in risk-adjusted mortality.

Nonetheless, concern over whether ICP-driven intervention induces morbidity exists. A group from the Netherlands compared the outcomes of two level 1 trauma centers which had different practice patterns in TBI management; broadly one used ICP to guide therapy, whereas the other used CT scanning.^12^ The centers were well balanced in terms of patient characteristics, with the ICP-guided group receiving more interventions than the other, without a demonstrable improvement in survival.

A larger analysis of the US National Trauma Databank (NTDB) compared patients with TBI who underwent ICP monitoring (n=708) with those who did not (n=938) and analyzed survival, after adjustment for confounders.^13^ ICP monitoring was associated with a 45% reduction in survival, which persisted when stratified for head abbreviated injury scale (AIS) scores. Several hypotheses have been postulated to explain these findings, from renal failure and pneumonia associated with aggressive fluid resuscitation through to the use of vasopressors stressing myocardium in pursuit of optimum hemodynamic performance. It should also be noted that the categorization of head AIS in the NTDB may not be sufficiently sensitive for a robust analysis.

Either way, such findings introduce equipoise into the debate, which has prompted the generation of prospective data in an effort to clarify the issue. The only randomized trial on this subject to date is the Benchmark Evidence from South American Trials:Treatment of Intracranial Pressure (BEST:TRIP) trial, published in 2012.^14^ This study was set in Bolivia and Ecuador and randomized 324 patients to receive guideline-based management for TBI either guided by ICP monitoring or imaging/clinical examination.

The primary outcome was a composite of survival time, impaired consciousness, and functional status at 3 and 6 months. Overall, there was no differences in primary outcome between the groups, although the ICP group required less brain-specific treatments (hyperosmolar fluids, hyperventilation, etc) (P=0.002). Interestingly, the distribution of serious adverse events was similar between the groups, suggesting that ICP monitoring alone was not driving overly aggressive intervention.

This trial has provoked a lot of controversy, but needs to be interpreted within the geographical context of the trial. As stated by the investigators, BEST:TRIP was, in fact, a trial of aggressive TBI management, guided by either assessment tool (ICP vs imaging/examination), which is a subtly different question from a head-to-head comparison of ICP monitoring versus no-ICP monitoring. The clinical trend to assess ‘bundles’ of care, rather than a single intervention, is becoming increasingly common in modern medicine and reflects the complexities of current therapies.

Furthermore, an understanding of the system of care within South America influences the interpretation of the study. Both the prehospital and post-ICU-discharge care lack some of the resources a comparable North American or European trauma system are able to deploy. It is conceivable that there were episodes of undocumented hypotension or hypoxia, which are known to influence outcome. Additionally, the high mortality post-ICU (day 14 onwards) may again reflect resource constraints which limit outcomes.

## Conclusion

Severe TBI constitutes a major source of trauma mortality and morbidity. The avoidance of extremes in blood pressure, oxygenation and carbon dioxide levels are critical to successful outcome. ICP monitoring remains a contentious subject with retrospective evidence demonstrating equipoise. The limited level 1 evidence available does not demonstrate harm or a survival benefit, but these findings are not easily generalizable to North American patients. It is unlikely that a further trial will be forthcoming, but ICP monitoring remains a recommended tool of TBI management.
